# Rapid and specific influenza virus detection by functionalized magnetic nanoparticles and mass spectrometry

**DOI:** 10.1186/1477-3155-9-52

**Published:** 2011-11-16

**Authors:** Tzu-Chi Chou, Wei Hsu, Ching-Ho Wang, Yu-Ju Chen, Jim-Min Fang

**Affiliations:** 1 Department of Chemistry, National Taiwan University, Taipei, 106, Taiwan; 2 Institute of Chemistry, Academia Sinica, Taipei, 115, Taiwan; 3 Department of Chemistry, National Central University, Jhong-Li, 320, Taiwan; 4 Department of Veterinary Medicine, National Taiwan University, Taipei, 106, Taiwan; 5 The Genomics Research Center, Academia Sinica, Taipei, 115, Taiwan

**Keywords:** Viruses, Influenza, Hemagglutinin, Magnetic nanoparticles, Mass spectrometry, Gel electrophoresis

## Abstract

**Background:**

The timely and accurate diagnosis of specific influenza virus strains is crucial to effective prophylaxis, vaccine preparation and early antiviral therapy. The detection of influenza A viruses is mainly accomplished using polymerase chain reaction (PCR) techniques or antibody-based assays. In conjugation with the immunoassay utilizing monoclonal antibody, mass spectrometry is an alternative to identify proteins derived from a target influenza virus. Taking advantage of the large surface area-to-volume ratio, antibody-conjugated magnetic nanoparticles can act as an effective probe to extract influenza virus for sodium dodecylsulfate polyacrylamide gel electrophoresis (SDS-PAGE) and on-bead mass spectrometric analysis.

**Results:**

Iron oxide magnetic nanoparticles (MNP) were functionalized with H5N2 viral antibodies targeting the hemagglutinin protein and capped with methoxy-terminated ethylene glycol to suppress nonspecific binding. The antibody-conjugated MNPs possessed a high specificity to H5N2 virus without cross-reactivity with recombinant H5N1 viruses. The unambiguous identification of the captured hemagglutinin on magnetic nanoparticles was realized by SDS-PAGE visualization and peptide sequence identification using liquid chromatography-tandem mass spectrometry (LC-MS/MS).

**Conclusions:**

The assay combining efficient magnetic separation and MALDI-MS readout offers a rapid and sensitive method for virus screening. Direct on-MNP detection by matrix-assisted laser desorption/ionization time-of-flight mass spectrometry (MALDI-TOF MS) provided high sensitivity (~10^3 ^EID_50 _per mL) and a timely diagnosis within one hour. The magnetic nanoparticles encapsulated with monoclonal antibodies could be used as a specific probe to distinguish different subtypes of influenza.

## Background

Influenza remains a major health problem for humans and animals. The recent cross-species transmission of avian influenza viruses to humans has raised a great concern for the possible global pandemic threat if the viruses become transmissible among humans.

Influenza viruses can be classified into types A, B and C. These subtypes are further designated according to the serological cross-reactivity of the antibodies against hemagglutinin (HA) and neuraminidase (NA), which are the most important glycoproteins on the surface of influenza virus with critical roles in virus infection and transmission. To date, 16 HA (H1-H16) and 9 NA (N1-N9) subtypes in influenza A viruses have been isolated from avian species. HA is translated as a single polyprotein, HA_0_, which exists in a trimeric assembly [1,2]. The transmembrane protein HA_0 _consists of two polypeptide chains, HA_1 _and HA_2_, linked by inter-chain disulfide bonds. For viral activation, HA_0 _must undergo an enzymatic cleavage to give two functional subunits, HA_1 _and HA_2 _[1,2]. Highly pathogenic avian influenza viruses, such as H5N1, contain many basic amino acid residues in the cleavage site of HA_0 _and are thus easily activated by trypsin and other proteases for systemic infection [1,2].

At present, four drugs are approved for influenza prophylaxis and treatment [3-5]: amantadine and rimantadine act as M2 ion channel blockers, and Tamiflu™ (the phosphate salt of oseltamivir) and Relenza™ (zanamivir) inhibit the activity of NA. For the most effective treatment, these anti-influenza drugs are recommended for use within 48 h of the onset of influenza symptoms because proliferation of the virus reaches a peak after 2 days of infection. Thus, timely and accurate diagnosis of specific influenza virus strains is crucial for effective prophylaxis, vaccine preparation and early antiviral therapy.

The detection of influenza A viruses is mainly accomplished using polymerase chain reaction (PCR) techniques or antibody-based assays to identify the relatively abundant nucleoproteins (NP) [6-13]. Because NP is only a type-specific protein, subtype- or strain-specific diagnosis cannot be achieved. For the specific detection of influenza viruses using real-time reverse transcription-polymerase chain reaction (rRT-PCR) [6-9], choosing proper primer pairs for subtyping becomes critical. Although sequence-based diagnosis often shows high sensitivity, the experimental procedures are tedious and may give false results.

According to a recent survey [8], the commercially available influenza diagnostic kits based on rRT-PCR can be used to detect H1N1 virus with a limit of detection in the range of 10^4.5^-10^5.5 ^TCID_50 _(50% tissue culture infective dose) per mL. However, a negative result does not rule out possible infection with influenza virus due to the overall low sensitivity (40-69%) of the diagnostic kits [8]. In contrast, an antigen capture immunoassay with specific monoclonal antibodies [10-13] is often utilized in rapid influenza diagnostic tests. An investigation into the commercially available test kits indicated that 10^4.7 ^mean embryo lethal dose (ELD_50_)/mL of avian influenza viruses in allantoic fluid can be detected by an antigen capture immunoassay [10].

The low sensitivity in antigen tests may be problematic in dealing with untreated samples due to nonspecific interactions with other proteins. The antigen-capture enzyme-linked immunosorbent assay (ELISA) has been explored to distinguish subtypes of influenza viruses with better sensitivity than immunoassays [11]. However, ELISA is time consuming and usually takes prolonged times (~ 12 hours) to provide results.

Alternative methods have been investigated for viral detection, including surface plasmon resonance [14], multiplexed flow cytometry [15], quartz-crystal microbalance [16], mass spectrometry [17-19], and microarrays [20-26]. With the power of peptide sequencing and database searches for unknown protein identification, however, mass spectrometry has been considered as one of the gold standard methods for protein analysis due to its low detection limit, rich structural information, and, most importantly, high accuracy. The Yip group was one of the first to integrate affinity capture techniques with direct mass spectrometric detection of target proteins from a complex mixture [27]. The concept was advanced further by Nelson and coworkers in the development of a mass spectrometric immunoassay (MSIA) using affinity pipette tips to selectively detect proteins and their variants [28-30]. Despite these existing methodologies, to our knowledge the application of affinity-based mass spectrometric methods for detection and identification of flu strains remains unexplored.

In combination with immunoassays utilizing monoclonal antibodies, mass spectrometry is especially useful for the identification of proteins derived from a target influenza virus. Mass spectrometry is not only applicable to confirm the subtype of virus but is also a powerful tool for the identification of the antigenic determinants on the viral HA [17-19]. Prior enrichment of the viral antigen is often utilized to improve the detection sensitivity and the coverage of peptide sequence identification in the mass spectra. An effective method for viral antigen enrichment using surface functionalized magnetic nanoparticles is pursued in this study.

Taking advantage of the large surface area-to-volume ratio and the unique chemical and physical properties of nanoparticles, a considerable number of studies on surface functionalization have been reported for biomedical applications. Among the various types of nanoparticles, magnetic nanoparticles (MNPs) have attracted increasing attention for the advantage of efficient separation from complex mixtures with a magnetic field [31-39]. This unique characteristic of MNPs surpasses traditional solvent intensive and time-consuming purification methods.

As demonstrated in our previous study [36,37], antibody-conjugated MNPs with proper surface protection act as efficient affinity probes for the rapid extraction of target proteins from human plasma. Because HA proteins are located on the surface of influenza viruses, MNPs modified with HA antibodies can be envisioned as an effective nanosensor for rapid detection of influenza viruses. Through this MNP-assisted mass spectrometry-based immunoassay, we expect to develop a simple and fast virus screening assay with unambiguous identification. H5N2, an avian influenza virus with low pathogenicity, and recombinant H5N1 pseudo-viruses were utilized as proof-of-concept model systems to evaluate the assay performance in terms of sensitivity and specificity. The data demonstrated the combined use of the antibody-MNP and MALDI-MS methods for the sensitive detection of influenza viruses and rapid screening of virus subtypes. The specificity of HA enrichment was confirmed by sodium dodecylsulfate polyacrylamide gel electrophoresis (SDS-PAGE) and peptide mass sequencing using liquid chromatography-tandem mass spectrometry (LC-MS/MS).

## Results and discussion

### • Preparation and characterization of ethyleneglycol-protected anti-HA antibody-conjugated magnetic nanoparticles

**Figure 1:**
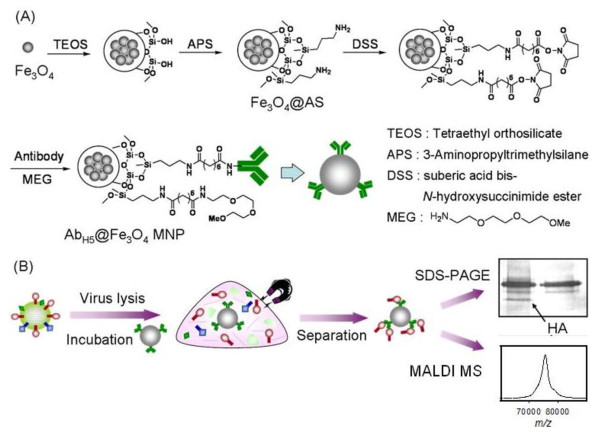
**Preparation of MEG-protected anti-HA antibody-conjugated magnetic nanoparticles for isolation and detection of HA proteins**.

The synthetic scheme for the antibody-conjugated MNPs is detailed in Figure 1A. The iron oxide nanoparticles (Fe_3_O_4_) were prepared by mixing FeCl_2 _and FeCl_3 _under basic conditions according to the previously reported procedure [40]. Through treatment with 3-aminopropyltrimethoxysilane (APS), the aminosilane coated MNPs (AS@Fe_3_O_4 _MNPs) exhibited an increased stability and contained amino groups for surface functionalization. The direct cross-linking of antibody with the AS@Fe_3_O_4 _MNPs was achieved through activation with a bifunctional linker, suberic acid bis(*N*-hydroxysuccinimide) ester (DSS), followed by incubation with the H5N2-specific monoclonal antibodies [41]. Compared with conventional approaches using protein G or protein A for antibody immobilization, direct conjugation was chosen to avoid non-specific association arising from protein A-conjugated MNPs. Because of the presence of other abundant non-antigenic proteins in the allantoic fluid, we noted that proper surface blocking of the MNPs was essential to avoid nonspecific interactions, which seriously compete with specific nanoprobe-virus recognition. Thus, further surface capping with an optimized concentration of methoxy-terminated ethylene-glycol amine (MEGA) was conducted to give the desired antibody-conjugated MNPs (designated as Ab_H5N2_@Fe_3_O_4 _MNPs). The MEGA-capped Ab_H5N2_@Fe_3_O_4 _MNPs were washed with phosphate buffered saline (PBS, pH 7.4) and stored at 4°C for months without loss of activity.

**Figure 2:**
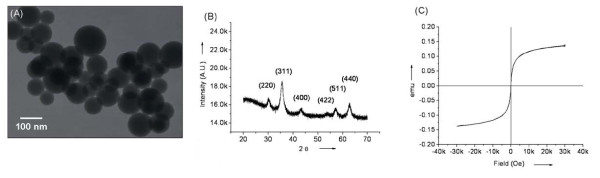
**Characterization of the aminosilane-coated iron oxide magnetic nanoparticles AS@Fe_3_O_4 _MNP**. A transmission electron microscopy image of AS@Fe_3_O_4 _MNP (A), structural characterization by powder X-Ray diffraction spectroscopy (B), and magnetization characterization by superconducting quantum interference device magnetometer (C).

The synthesized AS@Fe_3_O_4 _MNPs exhibited a spherical shape with an average diameter of ~90 ± 30 nm, as seen in transmission electron microscopy (Figure 2A). The spinel structure of AS@Fe_3_O_4 _MNP was revealed by powder X-ray diffraction (Figure 2B), which showed the characteristic pattern of diffraction peaks at (220), (311), (400), (422), (511) and (440) [42]. The AS@Fe_3_O_4 _MNP was superparamagnetic at room temperature (Figure 2C), as evidence by its hysteresis loop in superconducting quantum interference device magnetometer (SQUID) (*i.e.*, the magnetization reached saturation in an external magnetic field, but the magnetic character diminished in the absence of an external magnetic field). The unique superparamagnetic property of AS@Fe_3_O_4 _MNPs allowed easy magnetic separation from a complex mixture during synthesis or incubation.

### • Extraction of HA proteins by antibody-conjugated magnetic nanoparticles and on-bead MALDI-MS analysis

Our strategy for isolation and identification of HA proteins by Ab_H5N2_@Fe_3_O_4 _MNP is depicted in Figure 1B. Briefly, the Ab_H5N2_@Fe_3_O_4 _MNPs were incubated with virus lysate in a chosen buffer. After incubation, the newly formed complexes containing Ab_H5N2_@Fe_3_O_4 _MNPs and extracted HA (designated as HA-MNP complexes) were readily isolated by applying a magnet. The nonspecifically bound proteins were washed away, and the HA-MNP complexes were directly analyzed by either SDS-PAGE or MALDI-TOF MS. It was noted that both assays could be performed in an efficient manner without commonly required elution and desalting; our method thus reduced the time required for assay.

It was also noted that the incubation buffer was crucial to this experiment. Virus lysate in allantoic fluid has a complex composition, containing not only the H5N2 virus, but also other proteins abundant in the culture medium. Non-specific adsorption from these abundant proteins often interferes with immunoassays. Furthermore, HA is a trimeric transmembrane protein; thus detergent or salts are required to properly solubilize the hydrophobic protein and to avoid protein-protein aggregation. The effect of using different buffers on the specificity of affinity extraction was therefore evaluated. RIPA buffer, which is commonly used as a lysis buffer for immunoprecipitation assays, showed the best removal of abundant proteins capable of nonspecific interactions with Ab_H5N2_@Fe_3_O_4 _MNPs. Consequently, isolation of the viral HA protein by Ab_H5N2_@Fe_3_O_4 _MNPs was more efficient in RIPA buffer than in water or PBS buffer.

MALDI-MS can be combined with a biologically active probe to rapidly and specifically detect proteins of interest. To explore the capability of on-MNP readout by MALDI-MS, a protein pool mimicking a complex biological medium was prepared in 60 μL of PBS comprising various amounts of the recombinant HA protein of antigenic H5 type (200 ng, 100 ng and 50 ng) and a significant excess of other "nonantigenic" proteins such as transferrin (5 μg), fetuin (5 μg) and ribonuclease (1 μg). The abundance of the HA protein (1.8-0.5%, w/w) was purposely low to test the extraction efficiency of the Ab_H5N2_@Fe_3_O_4 _MNPs. After enrichment of HA proteins, the HA-MNP complex was mixed with sinapinic acid (SA), a MALDI matrix, and directly subjected to MALDI-MS analysis.

Although the antibody-antigen complexes have strong interactions with dissociation constants (*K*_d_) ranging from 10^-7 ^to 10^-11 ^M, most antibody-antigen complexes can still be dissociated at extreme pH (i.e., pH < 2 or pH > 12). The SA matrix solution used for MALDI-MS analysis has a low pH (< 2) and thus may directly elute the antigen bound to the antibody-conjugated MNPs on the MALDI sample plate.

**Figure 3:**
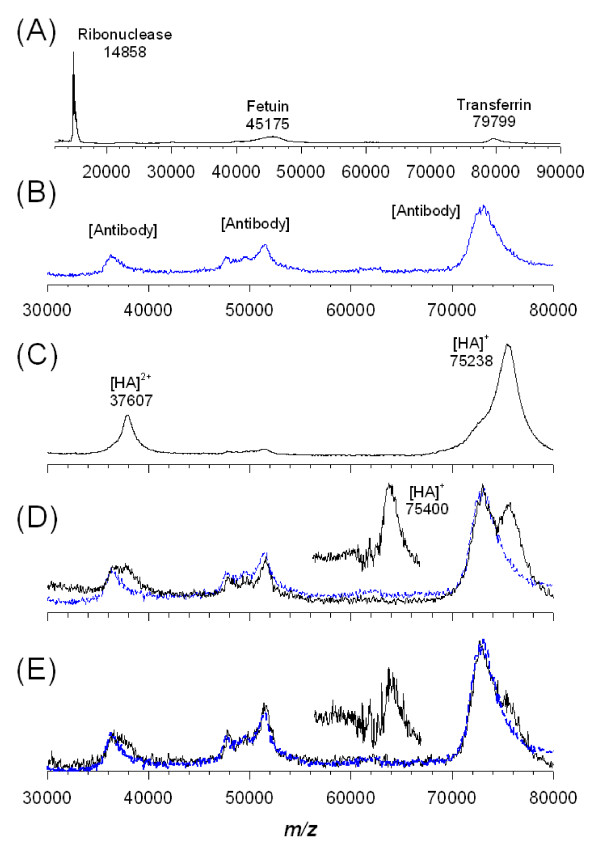
**Detection sensitivity of HA protein (H5 type) after extraction by Ab_H5N2_@Fe_3_O_4 _MNP**. MALDI mass spectra of (A) protein solution (60 μL) containing HA (200 ng), ribonuclease (1 mg), fetuin (5 mg) and transferrin (5 mg); (B) background peaks from antibody; (C) extraction by Ab_H5N2_@Fe_3_O_4 _MNP (HA signals at approximate 75 kDa and 37 kDa, corresponding to singly and doubly charged ions); (D) extraction from a protein solution (60 μL) containing HA (100 ng), ribonuclease (1 mg), fetuin (5 mg) and transferrin (5 mg) by Ab_H5N2_@Fe_3_O_4 _MNP; and (E) extraction from a protein solution (60 μL) containing HA (50 ng), ribonuclease (1 mg), fetuin (5 mg) and transferrin (5 mg) by Ab_H5N2_@Fe_3_O_4 _MNP. The arrow indicates the experimental *m*/*z *of HA. The blue dotted line indicates the antibody. The HA signals after subtraction of the antibody signals are shown in the insets of (D) and (E).

Prior to affinity extraction (Figure 3A), the MALDI spectrum of the protein mixture was complex and the targeted HA was not observed due to its low abundance and ion suppression. After affinity extraction (Figure 3B), two distinct signals appeared at *m/z *75238 and 37607 Da, which were derived from the glycosylated HA protein with one and two charges ([M + H]^+ ^and [M + 2H]^2+^, respectively) [25]. The characteristic broad peak shape of the glycosylated protein [43-45] could not be resolved under the mass resolution of our instrument. The shift of several kDa compared with the theoretical molecular weight of HA (theoretical average mass ≈ 64 kDa) [43-45] might be attributable to the extensive glycosylation of HA [25]. When the solution containing less HA (100 ng) was analyzed, the signal intensity significantly decreased and partially overlapped with the antibody signals to form a doublet mass peak (Figure 3D). After subtraction of the mass spectrum of the antibody (Figure 3B), the mass peak occurring at *m*/*z *~75400 Da clearly showed the presence of HA protein.

The affinity extraction of HA protein from a complex mixture was very efficient using the antibody-conjugated MNPs, as even a minute amount (50 ng) of HA protein was visible (Figure 3E). Assuming full recovery of all the HA protein (50 ng) present in the solution, the absolute detection limit was estimated to be 0.7 pmol (9 nM).

**Figure 4:**
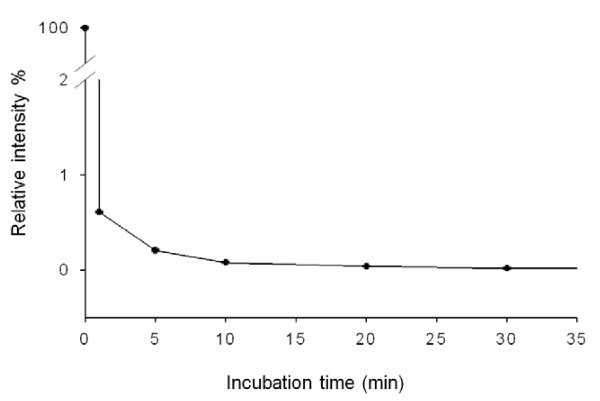
**Kinetic effect on HA enrichment using Ab_H5N2_@Fe_3_O_4 _MNP**. To investigate the time course of HA extraction, supernatant was sampled from a 60-mL reaction after different incubation times (1-30 min). HA remaining in the supernatant was vacuum dried and quantified by MALDI MS peak intensities.

To evaluate the efficiency of our detection method, we investigated the effect of incubation time on HA protein and antibody-conjugated MNP recognition. After incubation of antibody-conjugated MNPs with HA containing solution, the amount of remaining HA was concentrated and measured by MALDI-MS. The time course of such affinity extraction indicated that > 99% of HA protein in solution was captured by MNPs within 1 min (Figure 4). Thus, using antibody-conjugated MNPs for rapid and specific extraction, followed by direct mass spectrometric analysis for ambiguous readout, provides an efficient and accurate assay for HA protein.

### • Isolation of HA proteins from a virus sample for electrophoresis and mass spectrometric analyses

**Figure 5:**
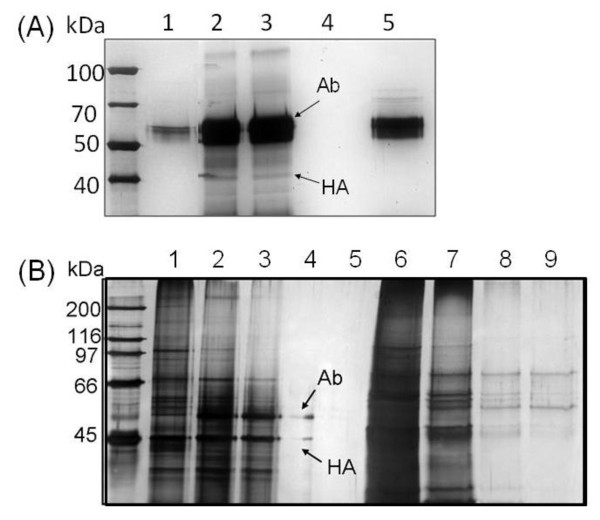
**HA purification performance by Ab_H5N2_@Fe_3_O_4_**. (A) Lane 1: H5N2 virus (200 ng) prepared from allantoic fluid; Lane 2: isolation of HA protein after incubation of Ab_H5N2_@Fe_3_O_4 _MNP with 1 μg of H5N2 virus lysate; Lane 3: isolation of HA protein after incubation of Ab_H5N2_@Fe_3_O_4 _MNP with 200 ng of H5N2 virus lysate; Lane 4: control experiment using MEG@MNP for incubation with 200 ng of H5N2 virus lysate; Lane 5: Ab_H5N2_@Fe_3_O_4 _MNP only. The antibody signals arise from dissociation of Ab_H5N2 _during SDS-PAGE analysis. (B) Purification efficiency of Ab_H5N2_@Fe_3_O_4 _MNP. SDS-PAGE patterns of Ab_H5N2_@Fe_3_O_4 _MNP before and after incubation of Ab_H5N2_@Fe_3_O_4 _MNP with H5N2 virus lysate in 2 × RIPA buffer. Lane 1: H5N2 virus; Lanes 2-5: incubation of Ab_H5N2_@Fe_3_O_4 _MNP (2 μL) with 1, 0.5, 0.1 and 0.01 μL of H5N2 virus, respectively; Lanes 6-9: supernatant of Lanes 2-5.

The affinity extraction of H5N2 viruses in allantoic fluid was also realized using Ab_H5N2_@Fe_3_O_4 _MNPs. As shown in SDS-PAGE analysis, without concentration of the virus lysate from allantoic fluid, only the abundant protein NP was observed in H5N2 virus (Figure 5A, lane 1). Using 2 × RIPA buffer, the Ab_H5N2_@Fe_3_O_4 _MNPs selectively isolated the viral HA protein from allantoic fluid (Figure 5A, lanes 2 and 3). As influenza HA protein (H_0_) is composed of two disulfide-linked polypeptides, HA_1 _and HA_2_, the use of a reducing reagent, like β-mercaptoethanol, in SDS-PAGE analysis will dissociate the HA protein into the two subunits HA_1 _and HA_2_. After treatment with β-mercaptoethanol, an electrophoresis band occurring at ~45-50 kDa was attributable to the glycosylated HA_1 _protein (Figure 5A, lanes 2 and 3) [25,43-45]. The band with molecular mass in the range of 50-60 kDa was ascribed to the proteins of antibody from Ab_H5N2_@Fe_3_O_4 _MNPs (Figure 5A, lane 5). As a negative control, we also synthesized MEG@Fe_3_O_4 _MNPs from aminosilane coated AS@Fe_3_O_4 _MNPs and MEGA without antibody conjugation. As expected, SDS-PAGE analysis showed that no viral protein was trapped by MEG@Fe_3_O_4 _MNPs. These results demonstrated the low background and the lack of false positives (Figure 5A, lane 4) in the presented method.

To evaluate the assay sensitivity, Ab_H5N2_@Fe_3_O_4 _MNPs (10 μg) were incubated with different amounts (0.05-1 μL) of the 1000-fold diluted H5N2 virus lysate (~10^7.8 ^EID_50_/mL, Figure 5B). As low as 0.1 μL of virus lysate, corresponding to ~10^3.8 ^EID_50 _of H5N2 viruses, was detected by SDS-PAGE using silver staining for visualization (Figure 5B, line 4).

**Figure 6:**
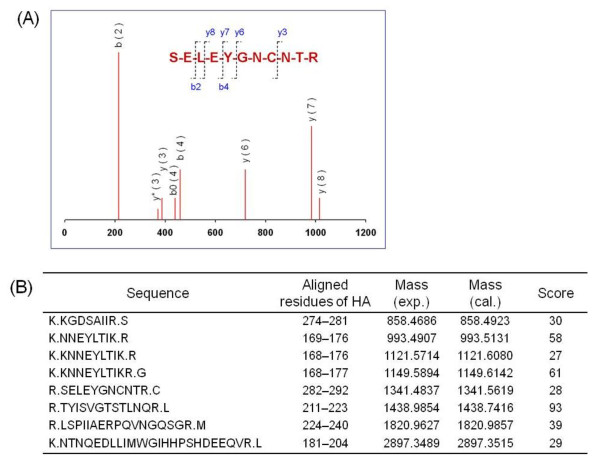
**The 45 kDa band in SDS-PAGE was identified as HA protein by LC-MS/MS analysis**. (A) An example MS/MS spectrum of the tryptic peptides at *m/z *1341.4837 from HA protein; the peptide fragments of y- and b-ions correspond to the sequence SELEYGNCNTR. (B) The experimental and calculated molecular masses and Mascot scores of the eight matched sequences.

To further confirm the identity of the captured protein, the electrophoresis band at ~45 kDa was digested with trypsin and subjected to LC-MS/MS analysis. Database searching with the Mascot engine confidently identified eight peptide sequences corresponding to the viral HA_1 _protein (Figure 6B). An example MS/MS spectrum is shown in Figure 6A for the peptide SELEYGNCNTR at *m/z *1341.4837, which corresponds to amino acids 282-292 of the HA protein from the H5N2 virus.

### • Differentiation of influenza virus subtypes

**Figure 7:**
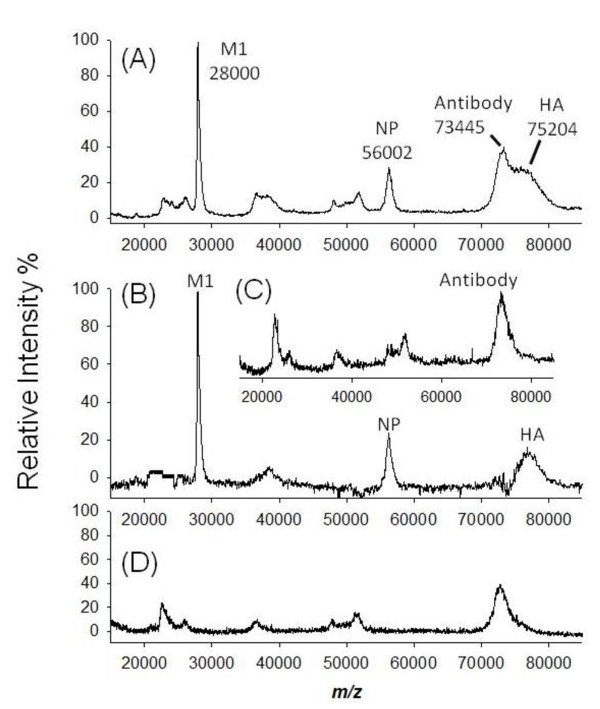
**Virus screening by incubation of Ab_H5N2_@Fe_3_O_4 _MNP with the lysate of different viruses**. The examined viruses (40 μL) include H5N2 (A/Duck/Taiwan/3233/04) and recombinant H5N1 (RG5, RG23, and NIBRG14). After incubation, only the H5N2 subtype was enriched. (A) HA, NP and M1 proteins were observed in the MALDI mass spectrum; (B) after subtracting the antibody signals (inset C), the HA, NP and M1 signals were clearly observed with good signal-to-noise ratio. For other viruses, only antibody signals were observed. A representative mass spectrum obtained from screening of RG5 is shown in (D).

Finally, we evaluated whether the high specificity of Ab_H5N2_@Fe_3_O_4 _MNP could be used to unambiguously differentiate virus subtypes. Besides the H5N2 virus (A/Duck/Taiwan/3233/04), three recombinant H5N1 viruses (RG5, RG23, and NIBRG14) were investigated. All of these influenza viruses belong to the H5 category of influenza A, but it was expected that the Ab_H5N2_@Fe_3_O_4 _MNP incorporating the monoclonal antibody specifically against the H5N2 virus would not have cross-reactivity with the H5N1 virus subtype. To validate such detection specificity, the lysates of H5N2 and H5N1 viruses were incubated separately with Ab_H5N2_@Fe_3_O_4 _MNPs in 2 × RIPA buffer at 25°C. After magnetic separation, the pellet was washed with 1 × RIPA buffer and subjected to MALDI-MS analysis. As shown in Figure 7A, the MALDI mass spectrum obtained from the on-MNP detection of extracted H5N2 virus (A/Duck/Taiwan/3233/04) revealed strong signals from viral proteins bound on Ab_H5N2_@Fe_3_O_4 _MNP. After subtraction of the antibody spectrum shown in Figure 7C, the mass spectrum clearly showed signals for glycosylated HA together with the relatively abundant NP (56002 ± 10 Da) and M1 (28000 ± 10 Da) proteins from H5N2 virus (Figure 7B). Gratifyingly, none of the recombinant H5N1 viruses were trapped by the Ab_H5N2_@Fe_3_O_4 _MNP. A representative mass spectrum obtained from a screening of H5N1 (RG5) is shown in Figure 7D, and only antibody signals were observed, confirming highly specific affinity isolation of H5N2. These data demonstrated the promising application of Ab_H5N2_@Fe_3_O_4 _MNPs to distinguish different subtypes in influenza virus surveillance.

## Conclusions

We have demonstrated a facile method for the detection of H5N2 influenza virus using aminosilane coated iron oxide nanoparticles with surface functionalization of monoclonal antibodies specific to the HA protein in H5N2. Through simple magnetic separation, the Ab_H5N2_@Fe_3_O_4 _MNPs showed effective isolation of H5N2 viruses from lysate for direct MALDI-TOF MS readout without the need for a tedious elution step. The detection limit was in the range of ~10^4 ^EID_50 _by SDS-PAGE or ~10^3 ^EID_50 _by MALDI-TOF MS. Our study demonstrated that the combined use of antibody-MNPs and MALDI-MS was suitable for sensitive detection of influenza viruses. Although the direct quantitative comparison of our method with other previously reported diagnostic methods is difficult because each method characterizes a specific property of the virus under various conditions, our present method appears to provide comparable or better sensitivity in the detection of influenza virus than commercially available kits [8,10]. These kits have a limit of detection in the range of 10^4.5^-10^5.5 ^TCID_50 _or 10^4.7 ^ELD_50_. Therefore, the method presented here can be utilized for the rapid screening of virus subtypes. The overall workflow of our method for influenza virus detection including virus lysis, magnetic separation and MALDI-TOF MS measurement can be routinely completed in one hour. We also demonstrated, for the first time, that the nanoprobe-based detection unambiguously differentiated the H5N2 virus from other closely related antigenic subtypes of (recombinant) H5N1 viruses in a highly specific manner.

With the automatic readout of MALDI-TOF MS, our method has the potential for integration into high-throughput virus assays, suggesting a promising application in early and accurate diagnosis of influenza viruses. Given the increasing use of virus screening assays, our current assay offers a flexible design for immobilization of other virus-specific molecular probes on a nanoparticle surface for diverse applications.

## Methods

### Materials

All of the chemicals were of reagent grade unless indicated otherwise. Ferrous chloride tetrahydrate (FeCl_2_^.^4H_2_O), ferric chloride (FeCl_3_), tetraethyl orthosilicate (TEOS), 3-aminopropyltrimethoxysilane (APS), 1-propanol, ammonia solution (28%), and dimethyl sulfide were purchased from Acros. Suberic acid bis-*N*-hydroxysuccinimide ester (DSS), 2-bromoethylamine hydrobromide (BEI), sodium carbonate, silver nitrate, α-cyano-4-hydroxycinnamic acid (CHCA), and sinapinic acid (SA) were purchased from Sigma-Aldrich. Formalin, methanol, acetonitrile (HPLC grade), and trifluoroacetic acid (TFA) were purchased from Merck. Acetic acid and sodium thiosulfate were purchased from J. T. Baker. All the chemicals were used as received without further purification. A homemade magnet was used in the separation of magnetic nanoparticles.

The HA proteins of H5 type were prepared using the H5 consensus sequence previously described [46]. The 1 × PBS buffer (pH 7.4, GIBCO) contained NaCl (137 mM), KCl (2.7 mM), KH_2_PO_4 _(1.5 mM) and Na_2_HPO_4 _(8.1 mM). The 1 × RIPA buffer (pH 8.0) contained Tris-HCl (50 mM, Amersham Bioscience), NaCl (150 mM), NP-40 (1%, Calbiochem), sodium deoxycholate (0.5%, Sigma) and sodium dodecyl sulfate (0.1%, USB). The 2 × RIPA buffer (pH 8.0) contained Tris-HCl (50 mM), NaCl (150 mM), NP-40 (2%), sodium deoxycholate (1%) and sodium dodecyl sulfate (0.1%). The TEN buffer (pH 7.5) contained Tris-HCl (50 mM, pH 7.5), EDTA (pH 8.0, 1.3 mM) and NaCl (100 mM).

### Instruments

Transmission electron microscopy (TEM) images were obtained on a Hitachi H-7100 Transmission Electron Microscope. Powder X-Ray Diffraction (XRD) was recorded by PANalytical X' Pert PRO. The magnetic effect was measured on a superconducting quantum interference device magnetometer (SQUID, Quantum Design MPMS7). LC-MS/MS spectra were recorded on a Waters Q-TOF™ Premier mass spectrometer (Waters Corp, Milford, MA). MALDI-TOF MS analyses were performed using an Applied Biosystems 4800 mass spectrometer (Applied Biosystems, Foster City, USA) equipped with a Nd-YAG laser (355 nm).

#### • Preparation of virus

The supernatant (0.2 mL/egg) of H5N2 influenza virus A/duck/Yunlin/04 (H5N2) was inoculated to the allantoic cavity of 9 to 11-day-old specific pathogen-free (SPF) embryonated hens' eggs, which were then cultivated in an incubator. The eggs that died within 24 h were discarded. Other eggs were incubated at 4°C for another 4 h to contract blood vessels. The allantoic fluid was then collected by needle and stored at -80°C.

To separate the cell lysate, the allantoic fluid was frozen and thawed several times to rupture the cells. The solution was subjected to centrifugation (3000 rpm, Centrifuge 5804R, Eppendorf, Germany) at 4°C for 15 min. The supernatant was treated with 1% BEI at 37°C for 18 h to inactivate the virus. After centrifugation (70, 000 × *g*, Avanti J-25 Centrifuge, Beckman) at 4°C, the virus pellets were collected, dissolved in TEN buffer, and stored overnight at 4°C. The virus was then separated by centrifugation (50, 000 rpm, Optima MAX-E Ultracentrifuge, Beckman) in a sucrose gradient (20-50%) at 4°C for 2 h. The concentrated H5N2 virus solution in TEN buffer was estimated to have an EID_50 _(50% egg infectious dose) value of about 10^10.8 ^per mL, corresponding to ~10^11 ^particles/mL estimated using the TEM-imaging of a mixture of viruses and a known concentration of polystyrene latex beads (137 nm in diameter) [47].

The recombinant H5N1 viruses RG5 (A/Anhui/1/2005), RG23 (A/turkey/Turkey/01/2003) and NIBRG14 (A/Vietnam/1194/2004) were obtained from Dr. Jia-Tsrong Jan (The Genomics Research Center, Academia Sinica) [46]. The TCID_50 _(50% tissue culture infectious dose) on Madin-Darby canine kidney (MDCK) cells was estimated to be around 3 × 10^6 ^(RG5), 1 × 10^5 ^(RG23), and 3 × 10^6 ^(NIBRG14) per mL, respectively, according to the Reed-Muench method [48].

#### • Preparation of antibody

The monoclonal antibodies against A/duck/Yunlin/04 (H5N2) virus were purchased from LTK BioLaboratories (Taipei, Taiwan) and purified on an immunoglobin affinity column. Briefly, a protein A HiTrap affinity column (Pharmacia biotech, Orsay, France) was activated with binding buffer (20 mM sodium phosphate buffer, pH 7.0). The ascites (1 mL) were mixed with Tris-HCl (200 μL of 1 M solution, pH 9.0) and applied to the affinity column. After the turbid impurities were washed off with binding buffer (10 × column volume), a striping buffer (700 μL of 0.1 M citric acid, pH 3.0) was applied to elute the antibodies. The collected antibody solution was then dialyzed in 1 L of PBS buffer (0.1 M) and stored at -20°C. The content of antibody protein (3 × 10^3 ^μg/mL) was measured by the Bradford method [49].

#### • Preparation of aminosilane coated iron oxide magnetic nanoparticles

Iron oxide nanoparticles (Fe_3_O_4_) were synthesized using FeCl_2 _and FeCl_3 _under basic conditions according to the previously reported method [40]. The freshly prepared Fe_3_O_4 _nanoparticles (30 mg) were suspended in 1-propanol (80 mL) and sonicated for 40 min at room temperature. Next, NH_4_OH (28% w/w, 8.94 mL), ddH_2_O (7.5 mL), and TEOS (0.1 mL) were slowly added to the above solution, and the mixture was stirred at 40°C for 2 h. APS (0.1 mL) was injected into the solution and the mixture was stirred for another 1 h. The blackish precipitates were collected with a magnet and washed with 1-propanol to give the aminosilane coated iron oxide magnetic nanoparticles (AS@Fe_3_O_4 _MNP).

#### • Synthesis of antibody conjugated magnetic nanoparticles

AS@Fe_3_O_4 _MNPs (0.5 mg) were suspended in DMSO (125 μL) and sonicated for 30 min at room temperature. DSS (5 mg) was added to the solution and the mixture was stirred at room temperature for 1 h. The precipitates were separated by magnet and washed with DMSO (250 μL) three times. The antibody (15 μL) was added at 4°C. After 1 h, a blocking reagent, 3, 6, 9-trioxadecylamine (a methoxy-terminated ethylene glycol amine, MEGA, 35 μL of 30 mM solution in DMSO), was added to the mixture. The mixture was stirred for another 18 h at 4°C, and the precipitates were collected by magnet. After washing with PBS, the antibody conjugated nanoparticles (Ab_H5N2_@Fe_3_O_4 _MNP) were re-dissolved in PBS (50 μL) and stored at 4°C. By a similar procedure but without the addition of antibody, the ethylene glycol encapsulated nanoparticles MEGA@Fe_3_O_4 _MNP were prepared and used as the control in experiments.

#### • Incubation of Ab_H5N2_@Fe_3_O_4 _MNP with viruses

Concentrated H5N2 virus solution (2 μL of 1.03 × 10^3 ^μg/mL in TEN buffer) was incubated with 2 × RIPA buffer (57 μL) at 25°C for 1 h. An aliquot (1 μL) of Ab_H5N2_@Fe_3_O_4 _MNP (0.5 mg in 50 μL of 1 × PBS) was added to the virus solution and incubated for 30 min. The HA-MNP complexes were then isolated by magnet and washed with 1 × RIPA buffer for the subsequent SDS-PAGE and MALDI-TOF MS analyses.

#### • SDS-PAGE analysis of HA-nanoparticle conjugates

The above-prepared HA-MNP complexes were resuspended in sample buffer containing 50 mM Tris-HCl (pH 6.8), SDS (1%, w/v), glycerol (10%, v/v), bromophenol blue (0.01%, w/v), and 0.7 M β-mercaptoethanol, and then heated at 95°C for 5 min. The sample was then separated on 10% SDS-PAGE gel in a running buffer containing Tris (0.3%, w/v), glycine (1.2%, w/v) and SDS (0.1%, w/v). Visualization of the protein bands was performed by silver staining. The SDS-PAGE was fixed with a solution containing 50% methanol, 12% acetic acid and 0.05% formalin for at least 2 h. The gel was washed three times with 35% ethanol for 20 min and soaked in a sensitizer solution (0.02% sodium thiosulfate) for 2 min. The gel was washed three times with deionized water and then incubated with a staining buffer containing silver nitrate (0.2%, w/v) and formalin (0.076%) in the dark for 20 min. The gel was then washed twice with deionized water. The band images were developed in a 50 mL solution containing sodium carbonate (6%, w/v), sodium thiosulfate (0.0004%, w/v) and formalin (0.05%). The silver reduction was processed for about 2 min until the expected intensity of protein was reached. Image development was terminated by treatment with a stop solution containing acetic acid (12%) and methanol (50%) for 5 min, then stored at 4°C in 1% acetic acid.

#### • Protein digestion and LC-MS/MS analysis

After electrophoresis, the band at ~45 kDa, corresponding to the HA_1 _protein, was taken from the SDS-PAGE gel and cut into small pieces (1 mm^3^). The gel slices were washed with 25 mM ammonium bicarbonate and 50% acetonitrile, reduced with 10 mM dithiothreitol, and then alkylated with 55 mM iodoacetamide. Trypsin (protein/trypsin = 20:1, g/g) was used to digest the protein by incubation for 12 h at 37°C. The resulting peptides were extracted with acetonitrile/TFA (50%/0.1%) and then acetonitrile. The peptide solution was concentrated on a SpeedVac and reconstituted in 8 L of 0.1% TFA aqueous solution (buffer A).

For protein identification by LC-MS/MS, the digested peptides were injected into a capillary trap column (2 cm × 180 μm) and separated on a BEH C18 column (25 cm × 75 mm × 1.7 mm, Waters ACQUITY, Milford, MA). The column was maintained at 35°C and the bound peptides were eluted with a linear gradient of 0-80% buffer B/A (buffer A, 0.1% TFA in H_2_O; buffer B, 0.1% TFA in acetonitrile) for 80, 120, 180, 210 and 270 minutes. MS was operated in ESI positive V mode with a resolving power of 10, 000. NanoLockSpray source was used for accurate mass measurement and the lock mass channel was sampled every 30 seconds. The mass spectrometer was calibrated with a synthetic human [Glu^1^]-Fibrinopeptide B solution (1 pmol/μL, from Sigma-Aldrich) delivered through the NanoLockSpray source. Data acquisition was operated in the data directed analysis (DDA) mode. The method included a full MS scan (*m/z *400-1600, 0.6 seconds) and three MS/MS scans (*m/z *100-1990, 1.2 seconds each scan) sequentially on the three most intense ions present in the full scan mass spectrum. The identification of peptide sequences was performed by database searching of MS/MS spectra using the Mascot algorithm (v2.1.0, Matrix Science, London, UK) against the NCBI database. The peak lists in the MS/MS spectra were extracted from Analyst QS 1.1 (Applied Biosystems) software with the default charge state set to 2+, 3+, and 4+. Search parameters for peptide and MS/MS mass tolerance were ± 0.3 and ± 0.1 Da, respectively, with allowance for two missed cleavages from the trypsin digest and variable modifications of carbamidomethyl (Cys) and oxidation (Met). Peptides were considered confidently identified if their Mascot individual ion scores were higher than the Mascot identity scores (*p *< 0.05).

#### • MALDI-TOF MS analysis

The sample of H5N2 virus enriched by trapping with Ab_H5N2_@Fe_3_O_4 _MNPs was mixed with fresh sinapinic acid (10 g/L) in a 1:1 (v/v) ratio. The complex was directly deposited onto the sample plate, dried in air, and then subjected to analysis on a 4800 MALDI TOF/TOF MALDI-TOF/TOF mass spectrometer (Applied Biosystems, Foster City, CA). To obtain a stable signal, a typical mass spectrum was constructed by averaging 1800 laser shots followed by noise reduction and Gaussian smoothing using Data-Explorer software (Applied Biosystems). The MALDI-TOF MS analysis was acquired with an Nd-YAG laser (355 nm) operating at a repetition rate of 200 Hz. The spectra were recorded in the linear mode using an accelerating voltage of 20 kV, a 19% grid voltage, a low-mass gate of 10 kDa, and a 630 ns delay time. The protein mixture of cytochrome *c *(12361 Da) and myoglobin (16952 Da) was used as an external mass calibration reference.

## Competing interests

The authors declare that they have no competing interests.

## Authors' contributions

YJC and JMF are the principal investigators and take primary responsibility for the paper. TCC synthesized magnetic nanoparticles with antibody conjugation and performed the magnetic separation of influenza virus. WH conducted SDS-PAGE and MALDI-TOF MS analyses. CHW instructed the preparation of monoclonal antibody and influenza virus samples. YJC instructed the biochemical analyses and wrote the manuscript. JMF instructed the chemical synthesis and also wrote the manuscript. All authors have read and approved the final manuscript.
